# Reflecting on Legal Responses to Intimate Partner Femicide in Scotland

**DOI:** 10.1177/10778012221094068

**Published:** 2022-08-22

**Authors:** Rachel McPherson

**Affiliations:** 3526 University of Glasgow, UK

**Keywords:** domestic abuse, femicide, intimate partner homicide, provocation, Scotland

## Abstract

This article considers legal responses to intimate partner femicide in Scotland. It reflects on how the plea of provocation on the basis of infidelity has been used, pointing to misrepresentation of the relationship between domestic abuse and stalking. From there, findings from 57 intimate partner femicide cases are presented. These findings indicate problematic stereotypes in how intimate partner femicide cases are reported and the operation of the “love narrative” in sentencing. It is concluded that more must be done to label intimate partner femicide cases appropriately and that improvements are achievable within the current criminal justice framework.

## Introduction

In recent years, there have been a number of significant studies that have interrogated the prevalence of intimate partner femicide (IPF)—that is to say the killing of a woman by her partner or ex-partner (Walklate et al., 2019). Other studies have examined how the criminal justice system understands and responds to IPF (Monckton Smith, 2021). However, there has been very little IPF research conducted in Scotland. This is significant since Scotland differs from the rest of the UK in two key respects. First, multiagency Domestic Homicide Reviews have not yet been implemented. A Domestic Homicide Review is a review of a death that follows domestic abuse. The aim of such reviews is to highlight to public bodies the ways in which responses to domestic abuse can be improved, and ultimately how such deaths can be prevented. Without such reviews, the difficulties associated with identifying IPF cases (Monckton Smith, 2021; Walklate et al., 2019) are exacerbated.^1^ The second way in which Scotland differs from the rest of the UK is with regard to the landscape of criminal defenses. In particular, the plea of provocation can be pled on the basis of infidelity. The operation of the plea on this basis impacts how IPF cases can be resolved in practice.

It is likely that both of these areas will be subject to reform: Discussion around the implementation of multiagency Domestic Homicide Reviews is already taking place and the Scottish Law Commission has recommended that provocation on the basis of infidelity be abolished (Scottish Law Commission, 2021). Against this backdrop, this article reflects upon Scotland's legal treatment of IPF cases, addressing a research gap in existing knowledge in a jurisdiction where there have been significant developments in responding to domestic abuse (Brooks-Hay et al., 2018; McPherson, 2021).

This article presents an analysis of existing case law on provocation on the basis of infidelity, pointing to how these cases have misrepresented the relationship between domestic abuse and stalking and have minimized the dangers women face upon the ending of a relationship. From there, findings from 57 IPF cases occurring between 2008 and 2019 in Scotland will be presented and analyzed. This will serve to add to the international evidence base on IPF which has been called for (Walklate et al., 2019) and will draw attention to the operation of violence against women myths in the reporting of IPF (e.g., men cannot control their anger; domestic abuse is a crime of passion; women would leave if it was so bad; it is probably not domestic abuse since all couples argue and fight) and the operation of the “love narrative” (Monckton Smith, 2021) in sentencing. The findings from this study indicate that although reform of the legal landscape is needed and welcomed, concerted effort will need to be made for the Scottish criminal justice system to respond to IPF cases appropriately.

## Homicide Statistics and Legal Responses to Domestic Abuse in Scotland

The most recent Scottish homicide statistics show that between 2010 and 2020 there were 162 female victims of solved homicide cases (Scottish Government, 2020a, calculated from Table 8). These victims account for approximately 23% of all homicide victims during the same period (Scottish Government, 2020a, calculated from Table 8). In keeping with international statistics on femicide (United Nations Office on Drugs and Crime [UNODC], 2018; Walklate et al., 2019), the largest single relationship between women killed and those accused of their killing their partner or ex-partner (43.8%; Scottish Government, 2020a, Table 8). The UN's *Global Study on Homicide* found that more than a third of the 87,000 women killed globally in 2017 were killed by an intimate or former partner (UNODC, 2018). The links between IPF and domestic abuse have long been recognized (Lees, 1997; Monckton Smith, 2021) as has the fact that in cases where there is coercive control and stalking, there is a higher likelihood that femicide will result (Dobash & Dobash, 2015; Monckton Smith, 2021; Proctor, 2017; Stark, 2009).

The introduction of Domestic Homicide Reviews in England and Wales in 2011 brought with it increased opportunity for information gathering about all forms of intimate partner homicide. Research findings published in 2016 indicated that 24 of 33 intimate homicide Domestic Homicide Reviews involved a perpetrator with a known history of violence (Home Office, 2016, p. 10). In nine cases, the perpetrator had a history of violence toward women. In six cases, four of which involved a female victim, the victim had a history of violence toward the perpetrator. The findings recognize that such violence may be retaliatory and that women are at an increased risk of death when they engage in such retaliatory violence toward an abusive partner (Home Office, 2016, pp. 10–11). As previously stated, Domestic Homicide Reviews have not yet been introduced in Scotland, exacerbating concerns regarding the identification and measurement of IPF (Monckton Smith, 2020; Walklate et al., 2019). In 2018, the *Equally Safe Strategy* (Scotland's national strategy for preventing and eradicating violence against women and girls) recommended that such multiagency reviews should be introduced in Scotland (Scottish Government, 2018). At the time of writing, criminal justice stakeholders such as Police Scotland are in discussion about their development and implementation.

Scottish legal responses to domestic abuse have developed significantly over the last 40 years, often as a result of committed feminist activism (McPherson, 2021). Following years of domestic abuse being categorized as other offenses such as assault, breach of the peace, or threatening or abusive behavior, the Domestic Abuse (Scotland) Act 2018 introduced a distinct, gender-neutral offense of domestic abuse toward a partner or ex-partner. Under this offense, harm can be either physical or psychological and can be committed either intentionally or recklessly (section 1(2)(b)). A leading expert on coercive control, Evan Stark, has described this approach to criminalizing coercive control as the “gold standard” (Scott, 2020). However, concerns about the problems of evidencing coercive and controlling conduct in practice have been raised given the continued existence of the formal requirement of corroboration in Scotland and the fact that this is a “course of conduct offense” (Cairns, 2020). Although the formal requirement for corroboration serves to render the Scottish landscape unique within the UK, evidencing domestic abuse has been recognized as a problem more widely (Bishop & Bettinson, 2018).

The landscape of civil protection orders in Scotland has also been subject to significant change since the introduction of the Matrimonial Homes (Family Protection) (Scotland) Act 1981. This Act was the first significant piece of private law introduced with the aim of offering protection to those experiencing domestic abuse. It introduced matrimonial interdicts (section 14) and exclusion orders (section 4), which could remove an abusive spouse from the matrimonial home, even where they were the owner of the property. Efforts to strengthen civil protection orders have since taken place through the introduction of the Protection from Abuse (Scotland) Act 2001, which allowed for a power of arrest to be attached to interdicts granted under the 1981 Act, and the Domestic Abuse (Scotland) Act 2011, which criminalized the breach of a domestic abuse interdict. The most recent development has come in the form of the Domestic Abuse (Protection) (Scotland) Act 2021, which introduced domestic abuse protection notices and domestic abuse protection orders. These provide short-term emergency measures to those suffering from domestic abuse in their home and aim to offer additional protection against the risk of homelessness.

Although Scottish legal responses to domestic abuse have developed significantly over the last 40 years, it has been pointed out that not all aspects of domestic abuse have been responded to equally. In particular, it has been said that there is a demarcation in how the Scottish legal system has responded to “non-fatal domestic abuse” and cases in which women kill a male abuser, with the latter experiencing legal inertia (McPherson, 2021). The subject of legal responses to IPF has been given less attention from a Scottish perspective but the analysis presented below would suggest that legal responses to IPF have similarly not developed in line with legal responses to domestic abuse more generally. In particular, the link between domestic abuse and IPF has often not been recognized.

## Provocation by Infidelity

Provocation is one of two partial defenses to murder, which is recognized under Scots law (the other being diminished responsibility). For provocation to be accepted there must be a recognized provocation. Two types of provocation are recognized in law: violence and infidelity. Where the plea is based on violence, there must be an immediate loss of control and the response must not be grossly disproportionate (*Gillon v. HM Advocate*, 2007). Where infidelity is the basis of the plea, there must be an immediate loss of control and the response must be that of an ordinary—rather than reasonable—person (*Drury v. HM Advocate*, 2001).^2^ The relationship must also be one in which there would be an expectation of fidelity (*McKay v. HM Advocate*, 1991). Concerns about provocation on the basis of infidelity are longstanding (McDiarmid, 2010; Wells, 2000) and reflect “an archaic approach arising from outdated concepts of male honour and sexual possession” (Scottish Law Commission, 2021, p. 147). Continued use of the plea in Scotland is also out of step with Scots law's approach to domestic abuse. Following recent judicial concern about the plea (*Donnelly v. HM Advocate*, 2017), the Scottish Law Commission stated in their discussion of the mental element in a homicide that they are inclined to recommend the abolition of this aspect of the plea (Scottish Law Commission, 2021). It is anticipated that there will be widespread support for abolition. Such a reform would bring Scotland into line with the rest of the UK while also marking an important turning point in understanding the reality of IPF. An analysis of provocation by infidelity cases illustrates how damaging the plea can be to the resolution of IPF cases, and how at odds this has been with Scottish responses to domestic abuse more generally.

In Scots law, the leading authority on both murder and provocation is *Drury v. HM Advocate* (2001). Here, the appellant sought to rely on provocation on the basis of infidelity. The facts of the case were that on 5 September 1998, Stuart Drury attacked and killed his ex-partner, Marilyn McKenna, outside her home with a claw hammer. Although Drury was ultimately convicted of McKenna's murder, the case continues to be recognized as deeply problematic. This discomfort was evidenced recently by its inclusion in the recent *Scottish Feminist Judgments Project* (Cowan et al., 2019). Claire McDiarmid (2010), who has previously written about the problems associated with provocation, submitted a feminist judgment and reflection on the case while Jay Whittaker used the case to create a powerful poem for the artistic strand of the project (Cowan et al., 2019).

At his trial, Drury had sought to rely on the partial defense of provocation on the basis that, at the time of the killing, it had become obvious to him that McKenna was engaged in a sexual relationship with another man. In his appeal against conviction, the court was asked to decide whether the proportionality of violence used in the fatal attack was relevant to the issue of provocation. The pathologist who examined McKenna said the facial injuries were the worst she had ever seen (*Drury,* at para 3). It was held that the trial judge had erred in his direction on proportionality and that there “is no hint in any of the authorities, nor would it be consistent with this concept, that there should be proportionality between the infidelity and the reaction to its discovery” (at para 7). The court was also asked to consider the suitability of the plea itself since it was contended that McKenna was not in a relationship with Drury at the time of her death. Having previously assaulted McKenna, Drury was now stalking her following their separation (Franchi, 2000). Numerous reports to the police had been made about Stewart Drury's behavior and in 1997 McKenna obtained an interim interdict prohibiting him from contacting her (Franchi, 2000). In the days before her death, McKenna telephoned her sister and said: “I’m going to be found in a pool of blood and everybody will be paying attention” (McCann & McKain, 2001). McKenna's family later described the case “as close to a text-book case [of domestic abuse] as it gets” (McCann & McKain, 2001).

In the appeal judgment, Lord Justice-General Roger noted that the advocate general put to the appeal judges that the trial judge was mistaken in accepting evidence that Drury and McKenna had a relationship that would give rise to a relevant plea of provocation. Of this matter, Lord Justice-General Roger stated:

We declined … to allow him to advance that submission, which came much too late and raised an issue that had never been put to the trial judge for his consideration, either during the trial or in the course of the appeal. We must therefore address the appeal on the footing that there was indeed evidence which would have entitled the jury, if they accepted it, to hold that the appellant could have expected sexual fidelity on the part of the deceased. The real area of dispute in the appeal related to the trial judge's direction on provocation. (*Drury,* at para 5)

Lord Justice-General noted that “some form of relationship had continued between the parties” (at para 2) during 1996 when they separated, and 1998, when she was killed, and that this relationship was of such a character that Drury “was justified in expecting [McKenna] to be faithful and that she had offered fidelity” (Lord Justice Cameron of Lochbroom, quoting the trial judge's charge at para 2).

This approach was consistent with previous authority on provocation by infidelity. In *Rutherford*, the court considered an appeal against a conviction for murder in circumstances where infidelity was alleged. In allowing the appeal against conviction and substituting the conviction for one of culpable homicide, it is commented:

The pannel was charged with the murder of a woman with whom he had lived as man and wife for a period of years. They had separated but were in touch with each other. The deceased informed the pannel that she had had an affair with another man with whom she had had sexual intercourse twice. She also indicated that she had been wanting to end that relationship. The pannel was stunned, upset and angry but did not react violently. Two days later the pannel met the deceased and on this occasion she told him that she had “been screwing that guy for months right underneath your nose” and that she had been enjoying every minute of it. There was no indication that the relationship was at an end. The pannel killed the deceased. (*Rutherford v. HM Advocate* 1998)

A reframing of separation is evident in both cases. In *Drury*, the refusal of the court to allow the Crown to contend with the assumption of an ongoing relationship sent a clear message to Scottish society: McKenna's experiences of domestic abuse were not central to her death. The social reality of McKenna's domestic abuse was denied and Drury's stalking of McKenna was reframed as evidence of a continuing relationship. There could be no greater misunderstanding of the context in which McKenna died.

The approach taken toward separation in *Drury* and *Rutherford* can be contrasted with Lord MacLean's direction to the jury in the earlier case of *McKean*—the only reported case concerning a female accused's access to provocation on the basis of infidelity. McKean had killed the male lover of her female partner, Connie Andrew, and sought to rely on the plea of provocation on the basis of infidelity in response to a charge of murder:

The more difficult question for you in this case, I suggest, is how far Miss McKean reasonably considered her relationship with Miss Andrew—which was undoubtedly homosexual—at the crucial time on Monday, 11 September 1995, to be a continuing one, with continuing obligations of faithfulness on the part of herself and Miss Andrew. They had, after all, been separated for about 10 days. (*McKean v. HM Advocate* 1997, at para 33)

*Drury*, *Rutherford*, and *McKean* represent three of 11 reported and commonly cited Scottish cases in which provocation on the basis of infidelity has been pled. Only one of these cases involves a female accused (*McKean*). This case of *HM Advocate v. Houghton* has previously been characterized as a case in which a female accused had a plea of provocation on the basis of infidelity accepted (Chalmers & Leverick, 2006), but an examination of note of appeal against sentence suggests that this is in fact a case in which the basis of the plea was violence, despite the reference to infidelity:

The appellant and the deceased had retired to bed but at this point, after the taunting, the appellant left the bed and made to push past the deceased, at which point he struck her on the mouth with his hand. Accordingly to Mr Shead who represented the appellant today that blow was accompanied by a threat to kill her. The appellant was then pursued through the house by the deceased who pushed her onto a settee. She then, in fear, went to the kitchen and took a sharp and thin boning knife, which she used in her job as a fish filleter. Mr Shead informed us that she did so because of her fear. She then went into the bathroom, followed by the deceased, who pinned her against the wall with his hand on her throat. At that point she struck the two blows which caused the deceased's death. (*Houghton v. HM Advocate*, 1999)

In eight of the reported cases referred to above, the plea was accepted (seven with a male accused and one with a female accused). These eight cases represent four IPFs and one further serious assault upon a woman (the remaining victims were male lovers of female partners). More recently, there have been three cases in which provocation on the basis of infidelity appears to have been pled: two with a female accused and one with a male accused. In the first case (*HM Advocate v. Grierson*, 2009), the plea was unsuccessful, and Grierson was convicted of murder. In the second (*HM Advocate v. Grant*, 2009) the plea appears to have been accepted. Catherine Grant pled guilty to the culpable homicide of Michelle Reid after Reid made a false claim that she was pregnant by Grant's partner, John Cameron. Cameron was placed on probation for three years for perverting the course of justice in the aftermath of the killing (BBC, 2009). In the final case, *HM Advocate v. Munro,* the plea was unsuccessful. In his sentencing, Lord Uist used the word “innocuous” to describe the photographs which led Munro to believe his partner had been unfaithful. This language made clear the court's view of the infidelity claim raised. In sentencing, it was also noted that Munro had previous convictions for violence against his ex-wife with Lord Uist commenting:

It is clear to me from that conviction and from the evidence which I have heard in the course of this trial that you are an evil and violent man. (*HM Advocate v. Munro*, 2013)

In this regard, Munro's claim was unequivocally rejected, and the narrative presented was one in which Munro was a threat to this woman and their child who was present in the house at the time of the murder.

Certainly, legal awareness of and responses to domestic abuse have changed in Scotland since *Drury*. Most significantly, stalking and domestic abuse are now both criminal offenses in their own right. However, in continuing to recognize provocation on the basis of infidelity, archaic, sexist attitudes toward women are upheld. Eradication of the plea would bring Scotland into line with other jurisdictions and would also bring Scotland into line with its own responses to domestic abuse elsewhere. However, to move on from a landscape in which domestic abuse was fundamentally misunderstood and mischaracterized for so long, there will need to be a concerted effort to make clear the link between IPF and domestic abuse and to respond to IPF cases effectively.

## Scottish IPF Cases

Fifty-seven IPF cases were identified as part of a larger study looking at Scottish homicide cases between 2008 and 2019. It is not suggested that these account for all IPF cases during this time, however, it is suggested that they are representative of Scottish cases during this period.

Thirteen of these cases were subject to appeal. Sentencing statements were available for 17 of the cases. Sentencing statements are made available on the Scottish Judiciary's website for a period of 12 months after the case. Older statements were obtained upon request to the Scottish Courts and Tribunal Service by the author. The remainder of the cases were identified using media reports. There is a history of using media reporting to identify cases that are “unreported”—that is to say, cases in which the legal judgment is not published (Gillespie, 1989; Moen & Shon, 2020; Sheehy, 2014). Key terms were used to search for cases through the search engine Google and LexisNexis News Library to identify relevant cases: terms such as “accused,” “arrested,” and “murder” were used alongside other terms such as specific defenses and specific High Courts in Scotland. The limitations of using media reports must be recognized, especially when trying to ascertain whether a case has been preceded by domestic abuse:

In news reports, it cannot be assumed that a failure to report a history of domestic abuse indicates that there was not one, or even that journalists were unaware of it. (Monckton Smith, 2012, p. 81)

Although corroboration of reporting sought to minimize the risks associated with this method of data collection, it is recognized that it is likely that most of these cases were preceded by male perpetrated domestic abuse, even where that was not explicitly reported.

### Overview of Cases

Of the 57 cases identified, 38 involved the death of a current female partner (66.7%) and 19 involved the death of an ex-partner (33.3%). Current partners have been broadly defined, and include consensual sexual partners, regardless of the length of time the parties were known to one another.

Fifty-two of the male accused identified in the study were accused of the killing alone (93%). Four were accused alongside another (7.1%): Three were accused alongside a single female and one was accused alongside a male and female. In keeping with what is known about intimate partner homicide generally, most killings occurred in a private residence (86%), most commonly a home shared by the accused and female victim (36.8%). A smaller number of fatalities occurred in outdoor spaces (10.5%).

Sixteen accused pled guilty to murder before trial (28.1%) and 11 accused resolved their case by submitting a guilty plea to culpable homicide (19.3%). Two accused (3.5%) were deemed to be unfit for trial due to their mental condition under section 53F of the Criminal Procedure (Scotland) Act 1995.

Twenty-eight trials resulted: 27 for murder and one for culpable homicide. Twenty-three men were convicted of murder (82.1%), one was convicted of culpable homicide (3.6%), one was fully acquitted (3.6%), one was convicted of a nonfatal offense (3.6%), and two were acquitted on the basis of mental disorder under section 51A of the Criminal Procedure (Scotland) Act 1995 (7.1%).

In total, therefore, 39 men were convicted of murder (68.4%) and 12 men were convicted of culpable homicide (21.1%), meaning that most men accused of IPF were convicted of homicide. Despite this high conviction rate, problems are evident in an analysis of these cases.

### Lack of Capacity Defenses

As suggested, two accused raised a 51A defense (acquittal on the basis of mental disorder) and two were unfit for trial due to their mental condition at the time of proceedings. Eleven accused advanced a defense of diminished responsibility, and this was accepted in seven of these cases, meaning that their conviction was for culpable homicide rather than murder. One of these cases involved assisted suicide in which the family was supportive of the offender's actions and there was significant medical evidence to corroborate the victim's deteriorating health. He was convicted of culpable homicide on the basis of diminished responsibility and admonished on appeal (*Gordon v. HM Advocate* 2018).

In *HM Advocate v. Buksh*, where diminished responsibility was accepted and a conviction for culpable homicide resulted, it was explicitly emphasized by the defense counsel that this was not a case in which domestic abuse had preceded the killing:

This is not a case where this man was a bad husband, there was no domestic abuse. This was an unhappy marriage, but however, unhappy it was it didn't justify killing his wife. He suffered an acute stress reaction. Three psychiatrists all agree that he was suffering from an abnormality of the mind (BBC, 2017).

Yet, the prosecutor's account to the court reveals that risk factors associated with IPF were present:

The accused and his wife had been married for 25 years, but over the last 10 or 11 years their relationship appears to have broken down and they became increasingly estranged, albeit, still residing under the one roof.

They slept separately and Mrs Buksh routinely slept on the sofa in the living room. Their children report that they barely spoke to each other.

Mr Lamont said the problems in the marriage intensified after Mrs Buksh went to Pakistan on holiday in May last year. He said she had become involved with another man and was making plans to move to Pakistan. (*HM Advocate v. Buksh*, 2017 as reported by BBC, 2017)

Elsewhere, in *HM Advocate v. Reilly*, another case in which diminished responsibility was accepted, the sentencing judge is reported as saying:

Social workers say that in their opinion you will pose a significant risk of harm to any future partners. You have two previous convictions for crimes of violence. (BBC, 2012)

In *HM Advocate v. Khan*, diminished responsibility was pled and evidence was led that the accused was suffering from an adjustment disorder at the time when he strangled his wife. In the sentencing, Lord Matthews noted:

There is no way of knowing whether the jury took the view that there was a murderous attack and that you were suffering from diminished responsibility, whether they took the view that the attack was not murderous but that you were fully responsible or whether they thought that the attack was not murderous and that that you were in addition suffering from diminished responsibility. The preponderance of the evidence as it was led before me, whatever may have been the situation at the earlier trial, leads me to the view that I should proceed to sentence on the last of these bases. (*HM Advocate v. Khan*, 2008)

Although domestic abuse was not referred to in the sentencing statement, the victim's family later reported their anger to the press, claiming that Iffat Kamal had been subject to “ritual of abuse” at the hands of Khan (Currie, 2009).

### Lack of Intention

Monckton Smith notes that the frequency of “lack of intention” defense positions in IPF cases. Lack of intention, as a defense, represents a claim by the accused that they did not have the *mens rea*—guilty mind—required for the offense to be satisfied. In Scotland, the *mens rea* required for murder is either wicked intention or wicked recklessness (*Drury*). Monckton Smith notes that a lack of intention defense can be a way through which the previous male perpetrated violence and control can be minimized and manipulated for the purposes of a defense (2021, p. 22). In the current study, this defense position was advanced in nine cases. In three of these nine cases, the claim that intention for murder was lacking manifested in the defense of accident specifically another defense which rests on the assertion that the *mens rea* of the offense in question has not been established. In eight of the nine cases in which lack of intention was pled, the accused was convicted of murder. As such, the defense was unsuccessful and rejected in most cases in which it was raised. Yet, even where a murder conviction resulted, problematic reporting was evident. For example, in *HM Advocate v. Shone* the couple's relationship before the homicide was described as “up and down” (BBC, 2014a). In *HM Advocate v. Buist* it was reported that the victim had previously phoned the police about a “domestic.” Buist is quoted as describing an “escalating row” and having “just lost the plot” in an attack that involved the victim being hit on the head with a brick and subjected to a 17 cm stab wound in the chest (BBC, 2014b). Judicial statements in the case of *HM Advocate v. Vita* suggest a centralizing of the victim's infidelity in a case in which the accused claimed his actions to be accidental:

You had an exemplary life but all that ended when you discovered she was having an affair with another woman. (BBC, 2014c)

Vita's sentence of life imprisonment with a punishment part of 12 years was subsequently appealed by the Crown for being too lenient, but this appeal was refused.

In two further cases in which lack of intention was pled, the accused claimed that the fatality had occurred during consensual “rough sex.” Feminist concern about this type of defense has been raised (Bows & Herring, 2020) and the “defense” has been the subject of attention following section 71 of the Domestic Abuse Act 2021 which prohibits the use of such a claim in murder trials in England and Wales. However, this provision does not extend to Scotland and there exists a precedent for Scottish juries returning a verdict of culpable homicide in circumstances where the accused claimed that the victim consented to strangulation during intercourse (*Rutherford v. HM Advocate*, 1947). In *Rutherford*, a finding of culpable homicide was made despite judicial warning that “the crime does not cease to be murder merely because the victim has consented, or even has urged the commission of the deed” (*Rutherford v. HM Advocate*, 1947). Although such a defense cannot align with the unequivocal common law position that a person cannot consent to their own physical injury (*Smart v. HM Advocate,* 1975), it has been suggested by the Scottish Law Commission that an equivalent provision to that in the Domestic Abuse Act 2021 may be beneficial in Scots law (Scottish Law Commission, 2021, p. 190), presumably as a way to reinforce the existing legal position.

In one of the cases in the current study where a defense of “rough sex” was advanced, the accused was convicted of murder. However, in *HM Advocate v. Bruce*, it was accepted that the strangulation occurred during intercourse and was part of “erotic sexual asphyxiation.” The victim's family campaigned against the use of the defense in this case, with BBC investigations illuminating previous statements made in court which were inconsistent with the acceptance of a guilty plea to culpable homicide on this basis:

After reading out sections of Mark Bruce's “Criminal Justice Social Work Report,” his defence lawyer stated that Bruce had told the authors of the report “there was no conversation between himself and Ms Miazek about violence during sex, there was no discussion and that at no point would she have expected such.”

The lawyer added: “In particular, Mr Bruce has stated in this report, or it is recorded in this report, that he accepts that he did not have the consent of the victim to use strangulation during sex.” (Bonnar, 2020)

It was also noted that another popular Scottish newspaper had used the headline “Strangled to death in kinky sex romp” to report the story (Bonnar, 2020), evidencing how IPF can be recharacterized and minimized, with the victim being portrayed as a contributor to their own death.

The sentencing statement for Bruce was requested by the author, but this was not among those provided by the Scottish Courts and Tribunal Service.

### Sentencing

Among the 39 men convicted of murder, the average punishment part of their life sentence (that is to say, the minimum period of punishment that must be served) was 17.2 years. The longest punishment part dispensed was 27 years and the shortest was 11 years. Of the seven men who faced imprisonment for a conviction for culpable homicide, the average sentence was 5.8 years. Eight men within the study group were subject to hospitalization/medical disposals as part of their sentence.

Of the 17 sentencing statements published, a small number made clear that the background was one of domestic abuse with two referring to the fact that the domestic relationship served as an aggravation to the offense. For example, in the sentencing statement for *HM Advocate v. Crossan*, Lady Scott noted that Crossan had killed his previous partner in 1999 and had another conviction for domestic violence against another victim. In the sentencing statement for *HM Advocate v. Rizzo* Lady Rae told Rizzo: “You clearly have a very concerning attitude towards women.” Twelve sentencing statements, including three where domestic abuse had been recognized, emphasized the cruelty of the murder.

In the majority of sentencing statements, the narrative presented was not one of domestic abuse and in four sentencing statements the relationship between the parties was characterized as loving, with empathy being shown toward the killer's distress:

The violence was initiated by her and she approached you with a knife.

You were clearly in love with [the victim] and would not have wished her any harm. It is a tragedy that you find yourself as a first offender in the High Court of Justiciary. These are all matters in your favour. (*HM Advocate v. Anderson*, 2016)

*

It is clear that you were unable to cope with the thought of separation and the change in your family circumstances which brought this about. I accepted entirely that you were upset and emotionally distraught as a result of the realisation that your partner no longer wished to live with you. (*HM Advocate v. Hetmanski*, 2011)

*

It is clear that you were distraught by the breakdown of your marriage and that you were having very great difficulty in coming to terms with it. (*HM Advocate v. Richardson*, 2010)

*

It appears that your actions … were the culmination of a lengthy period during which your anger at you wife having had the temerity to leave and divorce you have grown into a determination to make sure that she could not be free of you and live … in a sense you have also suffered loss through this death… (*HM Advocate v. Yazdanparast*, 2014)

Monckton Smith (2012) has previously emphasized the prevalence of the “love narrative” in the reporting of IPF cases, pointing to the fact that love, unlike hate, can be considered an acceptable motivation to kill. She also refers to observable violence against women myths perpetuated in the press to rationalize IPF: victim blaming, blaming outside influences such as alcohol, assuming that women lie about abuse, representing killers as unusual, and failing to acknowledge IPF as a common form of homicide, excusing male violence, reporting violence as an output of love and showing empathy with the killer's distress (Monckton Smith, 2012). These themes are evident in the press reporting related to the cases in this study while the “love narrative” is clearly evident in some sentencing statements. This is highly significant since it serves, inappropriately, to mitigate sentences and render men's actions reasonable and understandable, while distancing them from the reality of coercive control—a “pattern of oppression” (Stark, 2009, p. 4). As such, it recharacterizes the social reality in which women are killed most commonly.

## Discussion

More information is required about how many IPFs are committed each year in Scotland and the circumstances in which they arise. The introduction of Domestic Homicide Reviews is welcomed, but as Scotland prepares to enter a new landscape, understanding of domestic abuse will be key. Analysis of Domestic Homicide Reviews in England and Wales has already pointed to the importance of domestic abuse training for those involved in multiagency reviews of domestic homicide (Home Office, 2016, pp. 26–27). Such a training allows for agency workers to identify signs of domestic abuse and risk factors associated with homicide.

To date, no academic research has been carried out on public perceptions of domestic abuse in Scotland, but the Scottish Social Attitudes Survey suggests that most people do appreciate the wrongness of male-perpetrated physical abuse in intimate relationships (Scottish Government, 2020b). However, the results from this survey also suggest that verbal abuse is considered to be less serious, and that distinction is made between financial control and other monitoring behaviors such as multiple texting (Scottish Government, 2020b). Attitudes toward verbal abuse, physical abuse, and financial control have not significantly changed since 2014, but a change was evident in attitudes toward controlling behavior and wolf-whistling (Scottish Government, 2020b). It would seem that Scotland is continuing to move in a positive direction with regard to public understanding and attitudes. These have no doubt been informed, in part, by the recent criminalization of domestic abuse. However, nothing is known about attitudes toward IPF or cases in which women kill their male abusers.

The results from the 57 cases examined in this study show that the majority of men accused of IPF were convicted of murder. However, a closer examination of diminished responsibility cases suggests that there may be times when the return of a conviction for culpable homicide based on diminished responsibility has been inappropriate and has not taken into account the background of domestic abuse. Judicial responses and treatment of IPF cases suggest both increased awareness of IPF and adherence to violence against women stereotypes. Moving forward, there will need to be a concerted effort to recover from a landscape in which IPF has not formed part of the wider domestic abuse policy and in which the “love narrative” has prevailed: male violence has been excused in sentencing, violence is portrayed as an output of love and empathy is provided for the killer's distress. It should not be taken for granted that there will be understanding and awareness of the relationship between IPF and domestic abuse. One of the ways in which greater public understanding could be achieved is for the language of domestic abuse to be used explicitly in judicial statements. Such a language is notably absent from the materials examined. Even where sentencing judges have made clear that the context of the killing is one of the domestic abuse, the language of domestic abuse and/or coercive control is absent. It may be useful to look to the wider literature on criminalization on this point. The importance of “fair labeling” has long been recognized in theories of criminal law and criminalization (Chalmers & Leverick, 2008). It is recognized that, among other functions, the appropriate labeling of an offense communicates a clear message to society about the specific wrong which has taken place. Chalmers and Leverick comment that Scots law appears to formally endorse a “narrative approach” to labeling, given Schedule 3 paragraph 2 of the Criminal Procedure (Scotland) Act 1995, which holds that no label need be specified on an indictment (Chalmers & Leverick, 2008, p. 221). They question whether a narrative approach can constitute “labeling.” Broadly, they say, a description has been attached to an offender's behavior, but under a narrow definition, whereby both description and categorization are required, a narrative approach would not constitute labeling. They further distinguish between differentiation and description in labeling. For them:

To use a crude metaphor, differentiation refers to the box in which the offender is placed, while description refers to what is written on the side of the box. We do not mean to suggest that these two senses are entirely separable—in practice, for example, a description of conduct as manslaughter draws explanatory value from the fact that it has in some way been differentiated from murder. Ideally, however, and particularly in areas where the criminal law's structure may be less well understood by the general public, descriptive labels should be intelligible on a freestanding basis. (Chalmers & Leverick, 2008, p. 222)

It is not suggested that a separate offense of IPF be implemented in Scots law and this work is not intended to contribute to discussions about the appropriateness of the current structure of homicide (Horder, 2019), but it is suggested that reliance on the rationale of “fair labeling” may be helpful in this context and that specifically, IPF cases should be clearly linked with domestic abuse in both indictments and judicial sentencing statements. This would create opportunities for increased understanding and awareness of how often women are killed by their partners and emphasize the link between IPF and domestic abuse. The narrative approach toward labeling already in place in Scots law facilitates this as does section 1(5) of the Abusive Behaviour and Sexual Harm (Scotland) Act 2016 which provides that where an offense is aggravated by involving abuse of the partner or ex-partner of the person committing it, the court must state this. At times section 1 is referenced as an aggravating factor without explicit explanation as to what that means. Further explanation would help communicate the link between homicide and domestic abuse to a nonlegal audience.

Successful implementation across all relevant areas clearly depends on relevant relationships and domestic abuse being properly identified and recognized by police, prosecutors when marking up cases, juries (who may delete parts of the narrative), and judges who are in a position to communicate the facts and background of an offense through their public, published sentencing statements.

## Conclusion and Recommendations

This article does not offer an exhaustive account of IPF cases in Scotland. Instead, it has provided insight into how the Scottish legal system has responded to IPF, particularly over the last 25 years when there have been significant changes in how the Scottish legal system has responded to domestic abuse. The operation of provocation claims based on infidelity has contributed to a toxic environment in which domestic abuse and its dangers have been rendered invisible, misunderstood, and/or recharacterized. An analysis of recent IPF cases suggests that while most men accused of IPF are convicted of homicide, some convictions for culpable homicide may not be appropriate. Agencies, including policymakers, should also be aware of the link between domestic abuse and homicide because if the link between domestic abuse and homicide is clearly identified, it will support the need for resources for those experiencing domestic abuse and resources which might facilitate intervention before a homicide occurs.

Moving forward, in a justice system in which there is increased understanding of domestic abuse, there must be a commitment to a proper *description* of IPF. It must be embedded clearly within the language and context of domestic abuse to increase understanding and allow for a deeper understanding of this form of homicide. In order for this to happen, there must be a commitment to training within the criminal justice system.

As Scotland prepares to move into a new landscape where Domestic Homicide Reviews are implemented and provocation claims based on infidelity are potentially no longer accepted by the courts, knowledge, and understanding of domestic abuse will be vital. Several recommendations arise from the findings of the current study. These would serve to further improve legal responses to IPF during this period of evolution:

- Appropriate labeling of domestic homicides/IPF on indictments.
- Continued judicial training on the risk factors associated with IPF and how such risk factors should be regarded in sentencing.
- A continued commitment to referring explicitly to a background of domestic abuse during sentencing, if this is the relevant context.
- Appropriate and detailed data on how many homicides are preceded by domestic abuse in Scotland each year.

For Scotland to align with legal responses to domestic abuse more generally, a concerted effort will have to be made to ensure that the legacy of Scotland's treatment of and responses to IPF cases does not inform this future landscape. It is, therefore, with cautious optimism that a new era of legal responses to IPF is entered into.

## Notes

1. The Scottish Government present annual datasets on homicide and domestic abuse but, like many central bodies, do not draw connections between these two datasets. Eleven motives are included for homicide, including “rage or fury” and “jealousy” but not domestic abuse. Domestic abuse statistics make reference to homicide, but no further distinction is made between homicides with a male and female victim. The difficulties associated with data capture in this context are noted in this publication.

2. The reasonable person in law is the hypothetical person who represents the judgment of a typical member of the community. Reference to an ordinary person test is less commonly applied. A definition of the “ordinary person” was not provided in *Drury*, but it has been argued that the test is conceptually difficult since arguably, an “ordinary” person would not be provoked to kill (Tadros, 2007).
